# Objective Quantification Technique and Widely Targeted Metabolomics-Based Analysis of the Effects of Different Saccharidation Processes on Preserved French Plums

**DOI:** 10.3390/molecules29092011

**Published:** 2024-04-26

**Authors:** Shengkun Yan, Rong Dong, Jiapeng Yang, Guoqiang Wang

**Affiliations:** 1 Agricultural Mechanization Institute, Xinjiang Academy of Agricultural Sciences, Urumqi 830091, China; 2 School of Control Engineering, Xinjiang Institute of Engineering, Urumqi 830023, China

**Keywords:** French plums, preserved, weighted gene co-expression network analysis, widely targeted metabolomics, differential metabolic compounds

## Abstract

Vacuum saccharification significantly affected the flavor and color of preserved French plums. However, the correlation between color, flavor, and metabolites remains unclear. Metabolites contribute significantly to enhancing the taste and overall quality of preserved French plums. This study aimed to investigate the distinctive metabolites in samples from various stages of the processing of preserved French plums. The PCF4 exhibited the highest appearance, overall taste, and chroma. Furthermore, utilizing UPLC and ESI-Q TRAP-MS/MS, a comprehensive examination of the metabolome in the processing of preserved French plums was conducted. A total of 1776 metabolites were analyzed. Using WGCNA, we explored metabolites associated with sensory features through 10 modules. Based on this, building the correlation of modules and objective quantification metrics yielded three key modules. After screening for 151 differentiated metabolites, amino acids, and their derivatives, phenolic acids, flavonoids, organic acids, and other groups were identified as key differentiators. The response of differential metabolites to stress influenced the taste and color properties of preserved prunes. Based on these analyses, six important metabolic pathways were identified. This study identified changes in the sensory properties of sugar-stained preserved prunes and their association with metabolite composition, providing a scientific basis for future work to improve the quality of prune processing.

## 1. Introduction

Prunus (*Prunus domestica* L.) is a genus of plum in the rose family, originating from central and western Asia. It was later introduced to Europe and has since become extensively cultivated across Europe and the Americas. The US and Europe have been known as the main producers of plums, with the West Coast of the US, especially California, being a major production area, accounting for 93% of the total plum production in the US [1]. In China, plums are primarily grown in Shaanxi, Xinjiang, Gansu, Shanxi, and other regions. Recently, Xinjiang, particularly southern Xinjiang, has made significant efforts to introduce various plum varieties for cultivation. By 2022, Jiashi County of Kashgar, Xinjiang, had established a cultivated area of 450,000 mu for French plums, producing a total of 160,000 tons, representing 60% of China’s total plum output [2]. Jiashi County has emerged as the largest high-quality plum production base in China, and the prunes have been named French plums.

As a popular traditional food, dried prunes are not only favored for their unique flavor and color but also for their potential health benefits [3,4,5]. However, the changes in flavor, color, and metabolites of dried prunes during their conversion to dried fruit are complex and delicate, and these changes directly affect the sensory quality and nutritional value of the final product. And the pressurization technique is crucial in the preparation of preserved French plums, as it promotes the uniform penetration of sugars and flavor substances without significantly increasing the temperature, effectively retaining the nutrients and color of prunes while inhibiting microbial growth and ensuring food safety [6,7]. 

This study aims to investigate the key change mechanisms during the processing of preserved French plums in order to maximize the retention and enhancement of the original quality of prunes [8,9], which were found to be important for the enhancement of the taste and aroma of the dried fruit. Meanwhile, taking the change in color as the primary factor of consumers’ sensory experience, this study provided scientific guidance for obtaining an ideal product appearance by analyzing the patterns of the effects of different sugar penetration processes on color [10,11,12]. In addition, the results of metabolite analysis provided a new perspective to understand the biochemical changes during processing, which is crucial to enhancing the health value and market competitiveness of dried prunes.

Sensory evaluation and compositional changes in dried fruits as a result of different processing techniques have been widely reported [13,14,15,16]. However, previous studies have lacked objective evaluation indicators and analysis of integrated metabolites. The quantitative relationship between metabolite profiles and flavor and color is not known. With the increasing application of metabolomics in food analysis, the quantification of vast numbers of compounds in foods has become possible, and a high-throughput method of associating compounds with various sensory traits of foods is needed to screen for sensory quality-related compounds [17,18,19,20]. This study aimed to interpret the sensory changes and relevant metabolite conversions of French plums during the processing stages. A quantitative descriptive analysis was used for sensory evaluation, and widely targeted metabolomics was employed to analyze the metabolite compositions of French plums at different processing stages. The potential correlations between sensory features and metabolites were explored using weighted gene co-expression network analysis (WGCNA). The key metabolites were obtained from the highly correlated modules for further verification and interpretation. On this basis, KEGG pathway analysis of metabolites with significant differences was performed to provide guidance for improving the production process of prune fruit, improving product quality, and developing new products with specific health benefits.

## 2. Materials and Methods

### 2.1. Experimental Materials and Reagents

We purchased French plums (variety: *Franciscus*) from a market in Jiashi, Kashi, Xinjiang, China, in August 2023. We obtained methanol and acetonitrile from Merck (Shanghai, China) and formic acid from Aladdin (Shanghai, China). 

### 2.2. Processing of Preserved French Plums 

**Figure 1:**
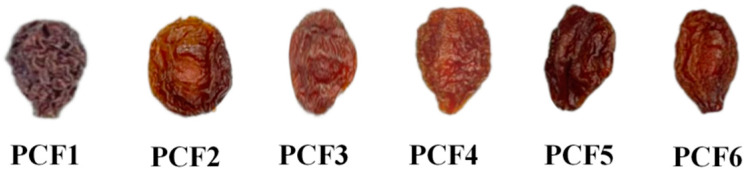
Pictures of preserved French plums at various stages: PCF1, PCF2, PCF3, PCF4, PCF5, and PCF6.

Dried French plums were softened at room temperature and then soaked at 80 °C for 15 min. After soaking, the plums were drained and cooled to room temperature to allow for sugar infiltration. Vacuum sugar permeation equipment (TN-50 type, Hefei Xiaoniu Light Industry Machinery Co., Hefei, China) was utilized to perform the sugar permeation treatment at different pressurization times. Once the osmosis process was completed, the sugar was drained, and the plums were washed, dried, and packaged. The treatment groups were as follows (Figure 1):

(1) PCF1: Fresh French plums were dried at 65 °C to obtain dried French plum samples.
(2) Sugar infiltration: PCF2: under usual atmospheric pressure, 70 °C constant temperature water bath for 1 h, sugar permeability at room temperature for 12 h; PCF3: at 0.07 MPa vacuum, 70 °C for 1 h, sugar permeability at room temperature for 12 h; PCF4: based on PCF3, a second vacuum sugar infiltration is performed; PCF5: based on PCF3, a third vacuum sugar infiltration is performed; PCF6: based on PCF3, a fourth vacuum sugar infiltration is performed.

The samples were placed in liquid nitrogen and freeze-dried in a freeze-dryer (Xinzhi Scientz-100F, Ningbo, China). The processing procedure was conducted in nine replicates, of which three replicated samples were used for sensory analysis, three replicated samples were used for the determination of color, and three replicated samples were used for metabolite analysis.

### 2.3. Sensory Analysis

The sensory evaluation team was composed of 10 food majors (5 males and 5 females) who had been trained in sensory analysis. They evaluated the color, fragrance, morphology, appearance, and taste, respectively. The sensory evaluation criteria were developed with reference to previous approaches [21,22]. A 100-point system was used, allocating 20% to sample appearance, 20% to color, 20% to fragrance, 20% to taste, and 20% to morphology (Table 1).

### 2.4. Determination of Color

Objective quantification indicators of color were measured three times using a CR-10 colorimeter (Konica Minolta, Tokyo, Japan). L represents the brightness of the sample’s appearance, a represents the red (+) to green (−) degree, and b represents the yellow (+) to blue (−) degree, respectively. The browning index (*BI*, Equations (1) and (2)) was used to describe the color change during drying [23].

(1) $$x=\frac{a+1.75L}{5.645L+a-3.012b}$$

(2) $$BI=\frac{100(x-0.31)}{0.17}$$

### 2.5. Sample Preparation and Extraction

#### 2.5.1. Dry Sample Extraction

The sample was placed in a vacuum freeze-drying machine (Xinzhi Scientz-100F, Ningbo, China) for lyophilization. The sample was then ground into a powder using a grinder (Sennheiser MM 400, Retsch, Haan, Germany) at 30 Hz for 1.5 min. Next, 50 mg was weighed out using an electronic scale (Mettler Toledo MS105DΜ, Greifensee, Switzerland), and 1200 μL of precooled (at −20 °C) internal standard extract consisting of 70% methanol in water was added. The extractant was added, ensuring that the addition speed did not exceed 50 mg/min. The mixture was vortexed once every 30 min for a duration of 30 s, repeating this action six times. The mixture was then centrifuged (12,000 rpm, 3 min), and the supernatant was aspirated. The supernatant was filtered through a microporous membrane (0.22 μm) and subsequently placed in the injection vial for subsequent analysis.

#### 2.5.2. UPLC Conditions

The UPLC-ESI-MS/MS system and the tandem mass spectrometry system were utilized for the analysis. The analytical conditions were as follows: an Agilent SB-C18 UPLC column (Santa Clara, CA, USA) was used; the mobile phase consisted of solvent A (water with 0.1% formic acid) and solvent B (acetonitrile with 0.1% formic acid). The specimen measurements were performed using a gradient program. Over 9 min, the program changed to 5% A and 95% B linearly, followed by 1 min at 5% A and 95% B. Then, it returned to 95% A and 5% B in 1.1 min and maintained this composition for 2.9 min. The flow rate was set at 0.35 mL/min, the column temperature was 40 °C, and the injection volume was 2 μL. The eluent was then connected to an ESI-Q TRAP-MS system.

#### 2.5.3. ESI-Q TRAP-MS/MS

The settings of the ESI were configured to a temperature of 550 °C, ion spray voltage (IS) at 5500 V/−4500 V, and gas pressures for ion source gas I (GSI), GSII, and curtain gas (CUR) at 50, 60, and 25 psi, respectively. 

The collision-activated dissociation (CAD) was set to high. MRM experiments with QQQ scans were performed using nitrogen as the collision gas at a medium setting. DP and CE for each MRM transition were customized. A specific set of MRM transitions was monitored during each elution period, corresponding to the metabolites identified at that time.

### 2.6. Statistical Analysis

Statistical analysis of the processing of preserved French plum experiments and the NVM tests were repeated three times. Significant differences in sensory quality and objective color indicators were assessed using a one-way analysis of variance (ANOVA; *p* < 0.05) using SAS 9.1.3 (SAS Institute Inc., Cary, NC, USA). The other statistical methods used are detailed below.

#### 2.6.1. PCA

Unsupervised principal component analysis (PCA) was conducted using the prcomp function in R (www.r-project.org (13 October 2023)). The data were scaled to have unit variance prior to performing PCA.

#### 2.6.2. Association of PCFs with Quality Objective Indicators via WGCNA

WGCNA, an advanced analytical method, was employed to decipher the relationships between gene expression profiles. This technique constructed a network reflecting the interconnectedness of genes through their expression levels across different samples, grouping genes with similar expression patterns into modules. WGCNA then correlated these modules with phenotypic traits, aiding in the identification of biologically significant pathways and genes associated with specific conditions or traits [24]. In this study, the WGCNA R package was used to analyze the French plum metabolite co-expression network, correlating preserved fruit quality indicators with natural variation in metabolites (NVMs) across different dark teas. The adjacency matrix was used to calculate the topological overlap measure (TOM) and to generate a cluster dendrogram. Metabolites were then clustered into color-coded modules representing distinct NVM categories using the DynamicTreeCut algorithm.

The WGCNA results were verified using PCA to examine the correlation between objective quantification indicators and PCFs within the key modules obtained through WGCNA. This analysis was performed to further identify characteristic PCFs related to color and taste objective indicators.

#### 2.6.3. HCA and PCC

The results of hierarchical cluster analysis (HCA) were visualized as heatmaps. Pearson correlation coefficients (PCC) were calculated using the cor function and visualized as heatmaps. Both analyses were conducted using the ComplexHeatmap package in R. In the HCA heatmap, a color gradient was used to represent the scaled metabolite signals of metabolites.

#### 2.6.4. Differential Metabolites

For the two sets of analyses, differential metabolites were identified using a VIP score greater than 1, a *p* value less than 0.01, and a fold change (FC) greater than or equal to 4.0 or less than or equal to 0.25. For multi-group analysis, differences were determined using the same criteria (VIP > 1, *p* value < 0.01, and FC ≥ 4.0 or ≤0.25) with the addition of ANOVA. The VIP values were obtained from the results of OPLS-DA. Prior to the OPLS-DA analysis, the data were log2 transformed and mean-centered. Permutation tests were performed using 36 permutations. Chiplot software (https://www.chiplot.online/) was used to visualize changes in the metabolites of French plums as heatmaps and volcano plots during processing.

## 3. Results and Discussion

### 3.1. Evaluation of Color Analysis and Sensory Quality in French Plum Samples’ Sugar Permeability at Different Conditions

#### 3.1.1. Color Analysis

**Figure 2:**
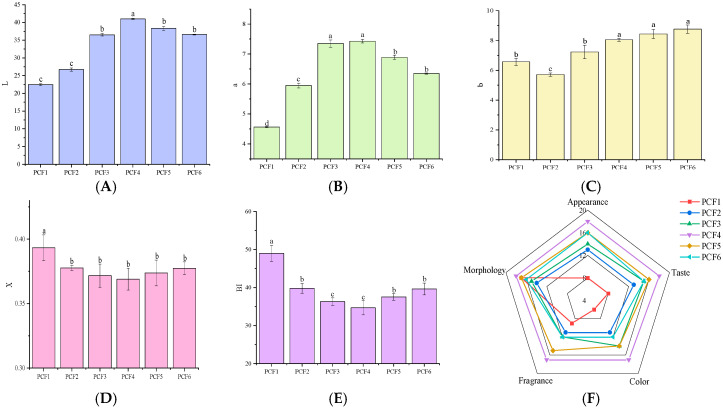
Chromatic difference analysis (**A**–**E**), and sensory qualities (**F**) of PCFs.

The color of preserved fruit is a direct measure of its quality and can significantly affect its market acceptance. As illustrated in Figure 2A–E, after saccharification treatment under various vacuum pressure conditions, the L value of the preserved fruit was highest in PCF4, indicating the surface color of the preserved fruit was the most transparent at twice the pressure times. As the pressure times increased, the L value decreased, and the surface color of the fruit darkened. This suggests that a low-pressure environment during vacuum saccharification might reduce water activity, thereby slowing down the Maillard reaction rate. However, as pressure times increased, the substrate concentration for the Maillard reaction also increased, accelerating the reaction. The trends of the a values were consistent with those of the L values, while the b values increased to varying degrees, suggesting that PCF4 maintained a good orange color. The trends of the x and BI values further supported these conclusions.

#### 3.1.2. Objective Quantification Indicators

Sensory evaluation is the most direct method of assessing food flavor. As shown in Figure 2F, different saccharification processes impacted the quality of the preserved fruit. In terms of color, PCF4 outperformed the other groups, with preserved fruit exhibiting uniform transparency, corroborating the colorimeter results. Regarding taste, PCF4 was superior as well, offering a balance of acidity and sweetness, suitable softness and hardness, and the distinctive flavor of French plums. PCF1 received a low score owing to its intense taste, but none of the six samples emitted any odor. In terms of appearance, except for PCF1, the skin was largely intact across all samples, and the fruits maintained their full shape. Microstructural examination revealed that increased pressure times led to more pronounced sand crystallization and sugar flow. The aroma scores across the six groups correlated with the color scores, with PCF4 having the most potent aroma. In terms of the total score, the ranking was PCF4 > PCF5 > PCF6 > PCF3 > PCF2 > PCF1. Overall, the sensory indices of PCF4 were significantly better than those of the other groups. Thus, from a sensory evaluation standpoint, a pressure frequency of two times was more suitable for processing French plums. Synthesizing the outcomes of the sensory evaluation, the PCF4 saccharification process demonstrated significant advantages across key sensory attributes, particularly in color, taste, and aroma. This not only highlights the effectiveness of the process in preserving the natural flavor and appearance of the preserved French plums but also reveals the importance of precisely controlling processing parameters to enhance consumer sensory experiences. These findings underscore the necessity for meticulous control over processing variables in food manufacturing and a deeper understanding of consumer preferences, providing a scientific basis for the production of prune fruit paste and pointing the way for future improvements in food processing and the development of new products. With further research and optimization, there is anticipation for the creation of an even wider range of high-quality foods that meet market demands and consumer expectations.

### 3.2. Analysis of PCFs in French Plum Samples’ Sugar Permeability at Different Conditions

#### 3.2.1. Full Mass Spectrometric Analysis and PCA of PCFs

**Figure 3:**
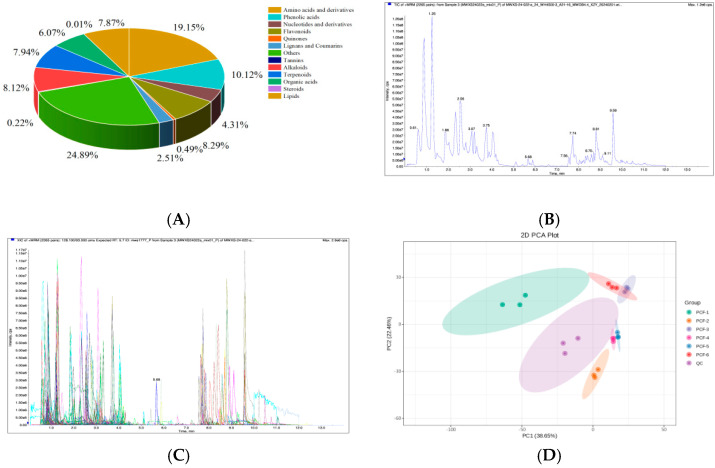
(**A**) Classes of metabolites identified in preserved French plums obtained through a widely targeted metabolic method. (**B**) Mass spectrometry detection of the total ion current in QC samples. (**C**) Multi-peak detection pattern of metabolites under the multiple-reaction monitoring mode. (**D**) PCA score scatter plot encompassing all metabolites.

The samples collected from various pressurization levels during different vacuum sugar penetration processes were subjected to metabolomics analysis. A comprehensive analysis identified 1776 metabolites (Figure 3A), encompassing diverse categories such as 344 amino acids and derivatives, 11 steroids, 99 lignans and coumarins, 189 terpenoids, 164 flavonoids, 230 phenolic acids, 55 nucleotides and derivatives, 171 lipids, 136 alkaloids, 96 organic acids, 17 tannins, 14 quinones, and 250 others. Two plots confirmed the data’s excellent reproducibility and reliability (Figure 3B,C). A non-supervised metrological PCA tool was utilized to incorporate QC samples. It was observed that the inclusion of QC samples facilitated the differentiation of PCF1 from the other five groups. Both PCF1 and PCF2 could be distinctly differentiated from the remaining four groups, suggesting that the non-volatile metabolites of the dried French plums and the ambient pressure osmosis sugar process were notably different from those of the other four sample groups (Figure 3D).

#### 3.2.2. Overall Difference Analysis of PCFs

**Figure 4:**
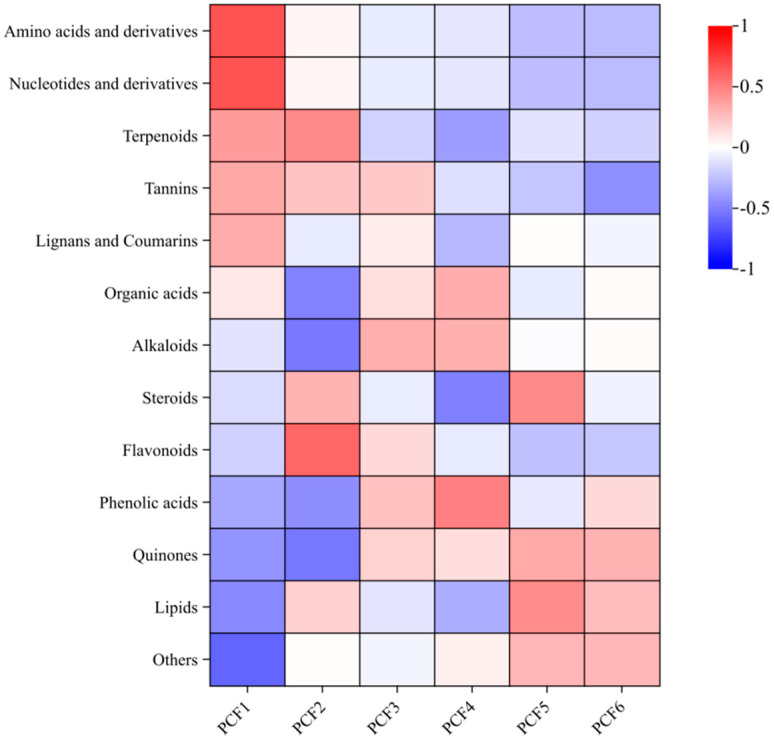
Heatmap of the variations in the component types for the various procedures. Note: “Others” included vitamins, sugars, ketone compounds, and stilbenes.

In all the preserved French plum samples, we observed significant changes in 13 types of substances (Figure 4). The contents of phenolic acids, lipids, and sugars in all preserved French plum samples were higher with the increase in pressure times. In PCF1, the contents of amino acids and their derivatives and nucleotides and their derivatives were the highest, and then these compounds declined with the increase in pressure times. The contents of alkaloids, organic acids, and flavonoids first rose and then decreased with the increase in pressurization times, reaching the peak value at PCF4. However, with the increase in pressure times, the quinone content first decreased and then increased. Phenolic acids are key substances that affect the flavor of French plums [25]. Vacuum saccharification may help reduce the oxidation and degradation of phenolic acids due to the low temperature and anoxic environment [26,27]. The vacuum environment also slows down the oxidation rate of lipids, and the increase in sugars is consistent with the purpose of the saccharification process, which is to increase sweetness and improve taste. Amino acids and their derivatives may decompose or change their structure to a certain extent during pressure osmosis, especially heat-sensitive amino acids such as lysine and arginine. Amino acids and sugars may undergo the Maillard reaction to form melanoidins and other macromolecular compounds [28], giving the French plums their characteristic brown color and flavor [29]. Alkaloids will degrade or transform when pressurized to a certain extent. Organic acids, which impart a sour flavor to the French plums, may migrate due to sugar penetration and have their stability affected by pressurization. However, excessive pressure or temperature may cause flavonoids to degrade.

During the processing, the effects of different numbers of pressures on the composition of substances showed that appropriate pressure conditions were essential for maintaining the flavor, nutritional value, and sensory quality of prunes. The contents of phenolic acids, lipids, and sugars increased with the increase in the number of pressures, while the contents of amino acids and their derivatives, nucleotides and their derivatives, alkaloids, organic acids, and flavonoids showed a tendency to increase and then decrease, which reflected the complexity of the stability of the substances during processing. In particular, the peak was reached under PCF4 conditions, which may indicate that this pressure condition provides an optimal balance in maintaining the sweetness, acidity, and health benefits of dried prunes. These findings emphasize the importance of precisely controlling pressure conditions to optimize the processing of dried prune fruits to enhance product marketability and consumer satisfaction.

### 3.3. Correlation Analysis of Differential Metabolites and Objective Indicators Based on WGCNA

**Figure 5:**
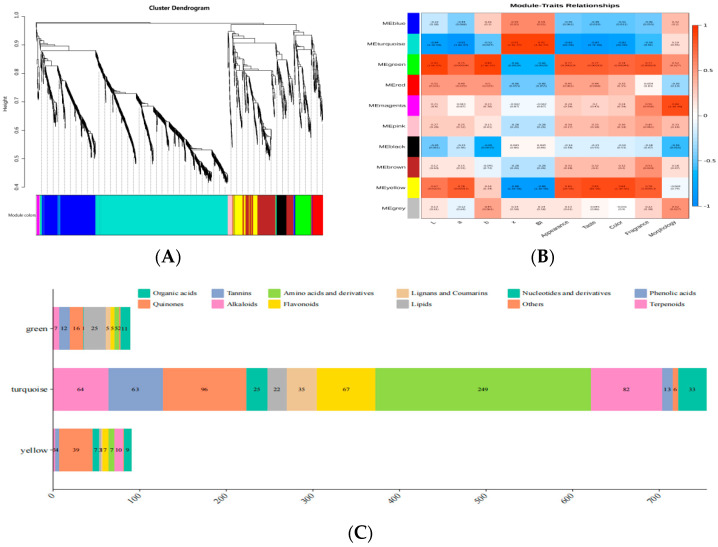
Clustering dendrogram of all metabolites for the identification of co-expression modules based on WGCNA (**A**). Module–trait relationships of all metabolites with color and taste objective quantification indicators based on WGCNA (**B**). Distribution of different types of metabolites in the 3 key modules (**C**).

Based on WGCNA, we established relationships between 151 metabolites and objective quantified indicators, including appearance color (L, a, b, x, and BI) and sensory evaluation index (color, fragrance, morphology, appearance, and taste). The WGCNA analysis generated 10 modules: blue, turquoise, green, red, magenta, pink, black, brown, yellow, and grey (Figure 5A,B). The turquoise module was positively correlated with x and BI values (*p* < 0.01) and negatively correlated with L, a, appearance, taste, and color (*p* < 0.01). In contrast to the turquoise module, the green module was positively correlated with L, a, b, appearance, taste, fragrance, and color (*p* < 0.01) and negatively correlated with x and BI values (*p* < 0.01). Magenta was positively correlated with morphology (*p* < 0.01). Black was negatively correlated with b (*p* < 0.01). Yellow was positively correlated with L, a, appearance, taste, fragrance, and color (*p* < 0.01) and negatively correlated with x and BI values (*p* < 0.01). The turquoise module showed the opposite trend to the green and yellow modules. The green, yellow, red, and pink modules were positively correlated with the appearance color index of preserved fruit and negatively correlated with BI. Conversely, the turquoise and black modules were negatively correlated with the appearance color index of preserved fruit. The results reflected the brightness and browning of French plums to some extent. Additionally, the appearance, taste, fragrance, and color of preserved fruit were consistent with the trend of appearance color and exhibited a partially different trend from morphology. As shown in Figure 5C, based on the key modules, 151 different metabolites were identified, including 36 amino acids and their derivatives, 13 phenolic acids, 9 nucleotides and their derivatives, 21 flavonoids, 1 quinone, 4 lignins and coumarins, 32 other classes, 2 tannins, 13 alkaloids, 9 terpenoids, 8 organic acids, and 3 lipids. Stress had a significant effect on most of the differential metabolites of preserved French plums’ seeds, so the flavor of preserved French plums in different groups was different. The analysis revealed significant correlations between the different modules and the brightness, browning, and sensory attributes of preserved French plums. In particular, the blue-green module was positively correlated with the brightness and browning of prunes, while the green and yellow modules were positively correlated with positive sensory quality attributes. These findings emphasize that the metabolite composition in preserved prune can be effectively modulated through precise control of processing conditions, such as pressure and temperature, to optimize their sensory quality and flavor properties. This study not only improves our understanding of metabolic changes during the processing of dried prune fruits but also provides valuable guidance to the food industry in developing dried fruit products that better meet consumer taste and quality requirements.

### 3.4. Analysis of Differential Metabolites’ Screening in Preserved French Plum Samples under Different Vacuum Saccharidation Conditions

#### 3.4.1. Analysis of Differential Metabolites by Volcanic Maps and Venn Diagrams

**Figure 6:**
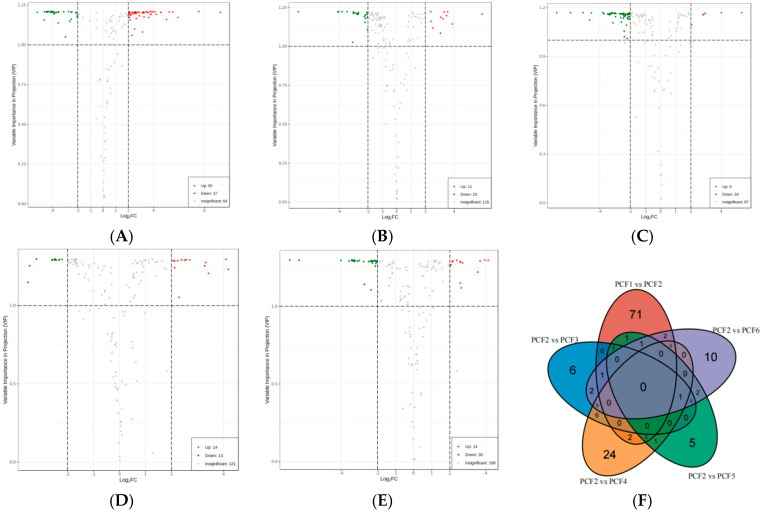
Volcano plot of the differential metabolites present in preserved French plum samples between PCF1 vs. PCF2 (**A**), PCF2 vs. PCF3 (**B**), PCF2 vs. PCF4 (**C**), PCF2 vs. PCF5 (**D**), and PCF2 vs. PCF6 (**E**). Venn diagram of the differential metabolites between PCF1 vs. PCF2, PCF2 vs. PCF3, PCF2 vs. PCF4, PCF2 vs. PCF5, and PCF2 vs. PCF6 (**F**).

To clarify the effect of pressure frequency on the sensory quality of prunus domestica, we used the criteria of an importance predictive value (VIP) > 1, *p* value < 0.01, and FC ≥ 4.0 or ≤0.25. Using comparison groups PCF1 vs. PCF2, PCF2 vs. PCF3, PCF2 vs. PCF4, PCF2 vs. PCF5, and PCF2 vs. PCF6, we analyzed the differential metabolites in three key modules. A total of 151 different metabolites were identified, of which 87 were in the PCF1 vs. PCF2 comparison group (Figure 6A, with 50 upregulated and 37 downregulated). There were 34 different metabolites in the PCF2 vs. PCF3 comparison group (Figure 6B, with 11 upregulated and 23 downregulated). There were 60 different metabolites in the PCF2 vs. PCF4 comparison group (Figure 6C, with 6 upregulated and 54 downregulated). There were 27 different metabolites in the PCF2 vs. PCF5 comparison group (Figure 6D, with 14 upregulated and 13 downregulated). The PCF2 vs. PCF6 comparison group had 44 differential metabolites (Figure 6E, with 14 upregulated and 30 downregulated). These data indicated that different pressure times had significant effects on the differentiation of preserved French plums.

The Venn diagram (Figure 6F) can be used to identify common and unique differential metabolites between samples of different French plums. Specifically, comparisons of PCF1 vs. PCF2, PCF2 vs. PCF3, PCF2 vs. PCF4, PCF2 vs. PCF5, and PCF2 vs. PCF6 revealed 151 differential metabolites, such as met-phe, ph-val-ser, kaempferol-3-o-rutinoside-7-o-glucoside, and so on. When comparing PCF1 vs. PCF2 and PCF2 vs. PCF6, we found that 3,4-dihydroxy-3,4-dihydro-2H-naphthalen-1-one, homogentisic acid*, xanthosine, and p-coumaric acid methyl ester are common differential metabolites between the two groups. 3,4-dihydroxy-3,4-dihydro-2h-naphthalen-1-one has strong stability and may contribute to the color and flavor of food. Homogentisic acid might help preserve the antioxidant properties of food and reduce flavor and color changes caused by oxidation. Xanthosine, a purine compound, could affect the flavor of food, and p-coumaric acid methyl ester has an important effect on the antioxidant properties and flavor of food. The results indicate that pressure can enhance the prominence of flavor substances in preserved Prunus fruit, and the color is more quantified with a slower oxidation rate.

**Figure 7:**
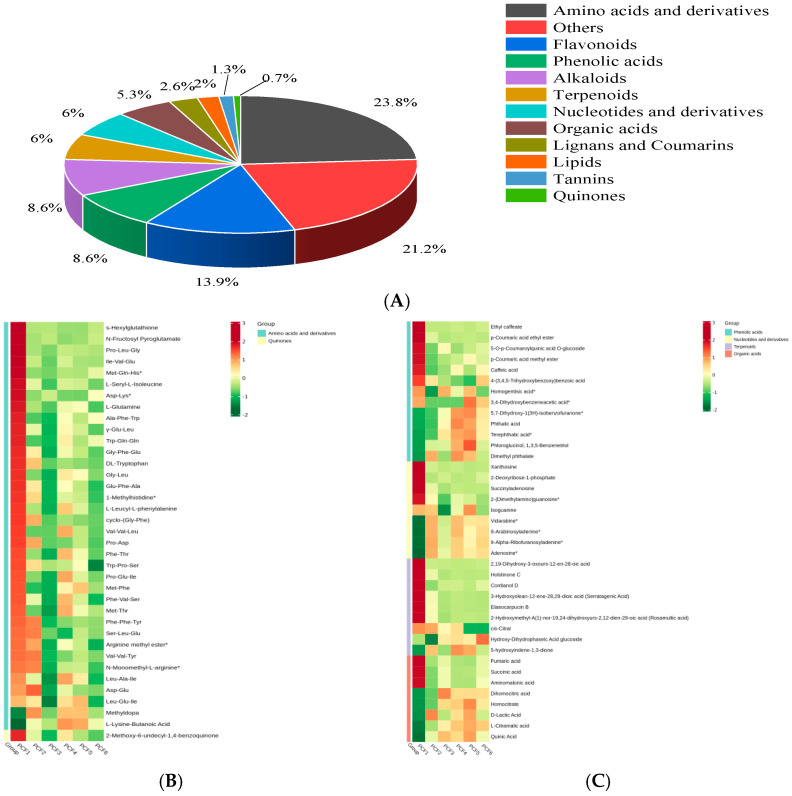

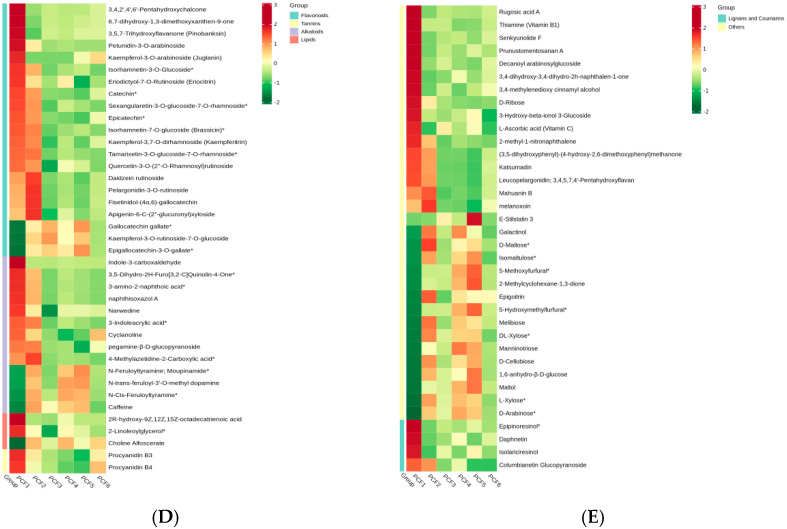
Pie chart showing 151 differential metabolites among all samples (**A**). Amino acids and their derivatives and quinones (**B**). Phenolic acids, organic acids, nucleotides, and terpenes (**C**). Flavonoids, tannins, alkaloids, and lipids (**D**). Others, lignin, and coumarin (**E**).

As shown in Figure 7A, the 151 differential NVMs included 36 amino acids and derivatives, 32 others (including sugars, ketones, aldehydes, alcohols, and vitamins), 21 flavonoids, 13 phenolic acids, 13 alkaloids, 9 terpenoids, 9 nucleotides and derivatives, 8 organic acids, 4 lignans and coumarins, 3 lipids, 2 tannins, and 1 quinone. To comprehensively analyze the changes in key differential metabolites caused by different processing methods, heatmaps of the 151 differential metabolites were obtained by classification (Figure 7B–E).

**Amino acids and derivatives.** Thirty-six amino acids and their derivatives exhibited highly significant changes after pressurized treatment (Figure 7B). Of these, 12 showed a significant decrease in content following pressurized treatment, namely dl-tryptophan, glu-phe-ala, 1-methylhistidine*, cyclo-(gly-phe), pro-asp, trp-pro-ser, phe-phe-tyr, ser-leu-glu, arginine methyl ester*, val-val-tyr, n-monomethyl-l-arginine*, and asp-glu, most of which do not directly enhance the flavor of the food. However, phenylalanine, phe-thr, pro-glu-ile, met-phe, phe-val-ser, met-thr, leu-ala-il, and l-lysine-butanoic acid increased significantly after the PCF4 treatment. These eight substances are dipeptides or tripeptides related to the freshness and flavor of food [30]. The increase in their content suggests that the freshness of preserved French plums improved and the taste was optimal after two pressurized osmosis sugar treatments. Compared with PCF1, there was no significant change in the content of amino acids and their derivatives after PCF6 treatment, which could be because the preserved fruit had already reached an equilibrium state post-pressurization. The PCF3 treatment might activate enzymes that promote the hydrolysis of amino acids, accelerating the hydrolysis process and leading to a decrease in amino acid content [31]. It can be seen that after two pressure saccharification treatments, the freshness of the preserved prune was improved and the taste reached its best.

**Others.** The other substances mainly included 11 sugars, 3 ketones, 2 aldehydes, 1 alcohol, and 2 vitamins (Figure 7C). The ketones, aldehydes, and alcohols are primarily related to aroma, and substances such as 5-methoxyfurfural*, 2-methylcyclohexane-1,3-dione, and 5-hydroxymethylfurfural* were significantly upregulated in PCF4 and PCF5. This indicates that twice-pressurized osmosis sugar and three-times-pressurized osmosis sugar treatments contributed to the accumulation of ketone-, aldehyde-, and alcohol-related aromas. The contents of manninotriose saccharides were upregulated in PCF4 and PCF5, contributing to the harmonization of the flavor of dried French plums. D-ribose, galactinol, D-maltose*, isomaltulose*, melibiose, DL-xylose*, D-cellobiose, 1,6-anhydro-β-d-glucose, l-xylose*, and d-arabinose*—10 saccharides in total—declined after pressure treatment. Pressure promotes saccharide degradation, as well as the Maillard and caramelization reactions, which contribute to the brown appearance and sweet or caramel aroma of preserved French plums [28]. These results indicated that pressure treatment promoted carbohydrate degradation, Maillard reactions, and caramelization reactions, contributing to the brown appearance and sweet charred flavor of prunes.

**Flavonoids and Alkaloids.** Flavonoids changed more significantly after pressurized treatment. Among them, petunidin-3-o-arabinoside, isorhamnetin-3-o-glucoside*, eriodictyol-7-o-rutinoside (eriocitrin), catechin*, sexangularetin-3-o-glucoside-7-o-rhamnoside*, epicatechin*, isorhamnetin-7-o-glucoside (brassicin)*, kaempferol-3,7-o-dirhamnoside (kaempferitrin), tamarixetin-3-o-glucoside-7-o-rhamnoside*, quercetin-3-o-(2″-o-rhamnosyl)rutinoside, daidzein rutinoside, pelargonidin-3-o-rutinoside, fisetinidol-(4α,6)-gallocatechin, and apigenin-6-c-(2″-glucuronyl) xyloside, a total of 14 substances, contributed to the darker hue of French plums, and reduced levels may cause the plums to become lighter in color (Figure 7D). Gallocatechin gallate*, kaempferol-3-o-rutinoside-7-o-glucoside, and epigallocatechin-3-o-gallate* showed upregulation after PCF3 and PCF5 treatments. These three substances were related to the yellow color of preserved French plums, suggesting that PCF3 and PCF5 treatments favored the accumulation of a yellow hue in the preserved fruits. When comparing the six groups of treatments, 13 alkaloid substances exhibited highly significant differences (Figure 7D). Of these, 3,5-dihydro-2h-furo [3,2-C]quinolin-4-one*, 3-amino-2-naphthoic acid*, naphthisoxazol a, 3-indoleacrylic acid*, cyclanoline, pegamine-β-d-glucopyranoside, and 4-methylazetidine-2-carboxylic acid* were downregulated after pressurized osmotic sugar treatment. N-feruloyltyramine, moupinamide*, n-trans-feruloyl-3′-o-methyl dopamine, N-Cis-Feruloyltyramine*, and caffeine were downregulated after PCF3 and PCF6 treatments. Alkaloids are related to bitterness [32], and the downregulation observed after pressurized treatment indicates that such treatment may reduce the bitterness in dried French plums. It can be seen from the above that the contents of various alkaloids are reduced after pressure saccharation treatment, which may indicate that pressure treatment can help reduce the bitterness in preserved prune fruits. This result has a positive significance for improving the taste and consumer acceptance of preserved prune fruits.

**Organic acids and terpenoids.** There were eight highly significant differences in organic acids (Figure 7E), of which three were downregulated compared to dried fruit (PCF1) in ambient pressure osmosis (PCF2) and pressurized osmosis treatments (PCF3-PCF6): fumaric acid, succinic acid, and aminomalonic acid. These may be lost from foods through osmosis in environments with high sugar concentrations. Elevated temperatures during the sugar permeation process can promote the degradation of these three organic acids or their reaction with other components, resulting in a decrease in their content [33]. Pressurized treatment leads to the upregulation of organic acids such as dihomocitric acid, homocitrate, d-lactic acid, l-citramalic acid, and quinic acid. Pressurized treatment may affect changes in enzyme activities and chemical reactions in preserved French plums, thereby promoting the activities of enzymes related to acid synthesis, such as homocitrate synthase, which increases the production of citric acid. In contrast, pressurized treatments may result in physical damage to French plums that may activate or alter intracellular metabolic pathways, affecting organic acid content [34]. The pressurized sugar infiltration method resulted in a sweet–sour balance in the dried fruit, which positively impacted the taste quality. There were nine highly significantly different substances among the terpenoids (Figure 7E), including 2,19-dihydroxy-3-oxours-12-en-28-oic acid, holstinone c, cordianol d, 3-hydroxyolean-12-ene-28,29-dioic acid (serratagenic acid), elaeocarpucin b, 2-hydroxymethyl-a(1)-nor-19,24-dihydroxyurs-2, and 12-dien-28-oic acid (rosamultic acid) with six substances in PCF1 and four in pressurized treatment. There was no significant difference in the groups, with two substances, hydroxy-dihydrophaseic acid glucoside and 5-hydroxyindene-1,3-dione, being upregulated after pressurized treatments and cis-citral being significantly downregulated after PCF5 and PCF6 treatments. Terpenes were mainly related to the aroma of dried French plum fruits, among which serratagenic acid and cis-citral contributed to rosy and lemony scents [35], respectively. However, cis-citral decreased after PCF5 and PCF6 treatments, suggesting that pressurized sugar infiltration three and four times was unfavorable to the accumulation of lemon aroma. In conclusion, pressure treatment technology has potential application value in the processing of preserved prune fruits, which can optimize the sweet–acid balance and aroma characteristics of preserved fruit by adjusting the content of organic acids and terpenoids so as to improve the overall flavor and sensory quality of the product.

**Phenolic acids and nucleotides and their derivatives.** Phenolic acids, nucleotides, and their derivatives were influenced by pressurized treatment. The treatment primarily affected 13 phenolic acids (as shown in Figure 7E), among which ethyl caffeate, p-coumaric acid ethyl ester, 5-o-p-coumaroylquinic acid o-glucoside, p-coumaric acid methyl ester, caffeic acid, 4-(3,4,5-trihydroxybenzoxy)benzoic acid, and homogentisic acid* decreased after the pressurized treatment. In contrast, 3,4-dihydroxybenzeneacetic acid*, 5,7-dihydroxy-1(3H)-isobenzofuranone*, phthalic acid, terephthalic acid*, phloroglucinol, 1,3,5-benzenetriol, and dimethyl phthalate increased after the treatment. The compounds 5-o-p-coumaroylquinic acid o-glucoside and homogentisic acid*, which are associated with bitterness, had their accumulation reduced by pressurized osmotic sugar treatment, thereby decreasing bitterness [36]. Homogentisic acid*, on the other hand, was differently affected by the number of pressurizations; three pressurizations reduced its content, while the remaining pressurizations had no significant effect. The analysis of nucleotides and their derivatives revealed that the levels of xanthosine, 2-deoxyribose-1-phosphate, succinyladenosine, and 2-(dimethylamino)guanosine* remained unchanged between pressurized and atmospheric pressure osmotic sugar treatments. The contents of vidarabine*, 9-arabinosyladenine*, 9-alpha-ribofuranosyladenine*, and adenosine* increased in preserved French plums under different osmotic treatments compared with those in PCF1. However, the levels of these four substances were not significantly affected by the sugar permeation processes. 9-arabinosyladenine, 9-alpha-ribofuranosyladenine, and adenosine, all adenine derivatives, along with adenosine, are considered key ingredients in enhancing the freshness of food. These nucleotides, together with inosinic acid and guanosinic acid [37], work synergistically with glutamic acid to significantly enhance food freshness, which is recognized as the sixth flavor, in addition to the traditional five basic tastes: sweet, sour, bitter, salty, and umami. Adenine not only enhances the flavor of food but also improves its texture, providing a sense of richness and fullness that makes the food more satisfying to the palate. This aligns with the results of sensory evaluation. Xanthosine mainly affects the color, aroma, and astringency of dried French plum fruits [38]. Compared with untreated dried French plums, various osmotic sugar treatments decreased the xanthosine content, which suggests that such treatments can reduce the astringency of French plum products. It can be seen from the above that the flavor characteristics and sensory quality of preserved fruit can be optimized by adjusting the content of phenolic acid and nucleotide so as to improve the overall flavor and consumer acceptance of the product.

#### 3.4.2. Metabolic Pathway Analysis

**Figure 8:**
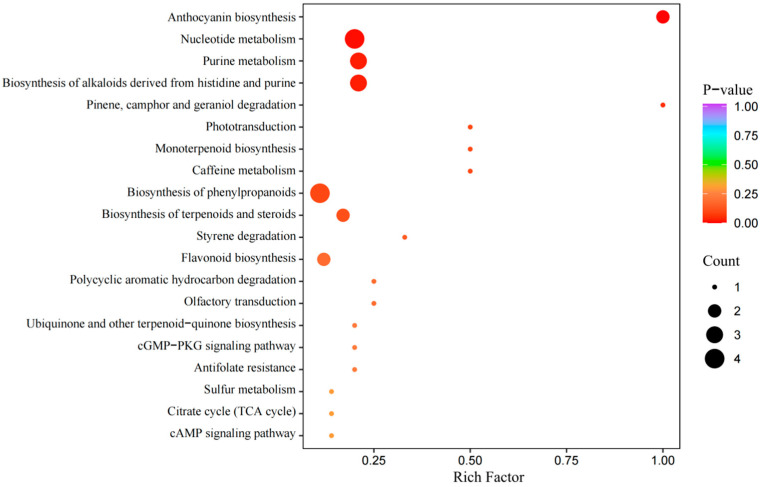
The KEGG-rich gas bubble diagram of different processing conditions (*p* value Top 20).

Figure 8 shows the enriched bubble plots of the metabolic pathways under different processing conditions. All the differential metabolites in the group were matched with the KEGG database to obtain information on the pathways involved in the metabolites. There are six significant metabolic pathways: nucleotide metabolism, purine metabolism, biosynthesis of alkaloids derived from histidine and purine, biosynthesis of phenylpropanoids, and anthocyanin biosynthesis. The specific analysis is shown below. 

The nucleotide metabolism recognizes that nucleotides are not only components of intracellular signaling molecules but also act as natural flavor enhancers in foods to enhance the freshness of preserved prunes. The overall texture and flavor of dried fruit can be enhanced by controlling conditions during processing to maintain or increase the levels of specific nucleotides. In addition, changes in nucleotide metabolism may affect the synthesis of other flavor-related compounds, such as the metabolism of certain amino acids, which in turn affects the overall flavor profile of preserved French plums.

In purine metabolism, the concentration and type of purine metabolites in foods may affect the flavor and nutritional value of preserved French plums. For example, uric acid, the end product of purine metabolism, may affect the sensory quality of the food in some cases. By optimizing processing conditions, the accumulation of undesired metabolites may be reduced while retaining or increasing compounds that are beneficial to flavor and nutrition.

Alkaloid biosynthesis derived from histidine and purines through alkaloidal compounds may have specific physiological activities in preserved prunes, such as antioxidant, anti-inflammatory, or other pharmacological effects. By controlling the synthesis of specific alkaloids during processing, additional health benefits may be conferred on preserved French plums, increasing the market appeal of the product.

In phenylpropane biosynthesis, phenylpropane metabolites such as flavonoids and lignans have a significant impact on the color, texture, and nutritional value of dried prunes. Flavonoids not only provide health benefits but may also affect the color and antioxidant properties of dried fruit. Lignin synthesis and deposition, on the other hand, affect the structure and texture of dried fruit. By regulating the synthesis of these metabolites, the appearance and texture of preserved French plums can be improved while maintaining their nutritional value.

Anthocyanins, as natural pigments, play a decisive role in the color of preserved French plums. By controlling the environmental factors (such as light, temperature, and nutrient supply) that affect anthocyanin synthesis during processing, the color of dried fruit can be optimized to make it more vibrant and attractive. Meanwhile, the antioxidant properties of anthocyanins also help to maintain the nutritional value and prolong the shelf life of preserved French plums.

In summary, by deeply understanding and regulating these metabolic pathways, precise control of flavor, color, nutrition, and health benefits can be achieved during the preparation of preserved French plums. This will not only help to improve the sensory quality of the product but also satisfy consumer demand for healthier food products, thereby gaining an advantage in the highly competitive food market.

## 4. Conclusions

This study investigated the metabolite profiles of French plums processed in different ways and their associations with sensory characteristics, including color and taste. During the processing of French plums, the color becomes brighter, sweetness is enhanced, astringency and browning are reduced, and the form becomes fuller. The metabolites in French plums were determined by widely targeted metabolomics, identifying 1776 metabolites classified into 13 classes (amino acids and derivatives, nucleotides and derivatives, terpenoids, tannins, lignans and coumarins, organic acids, alkaloids, steroids, flavonoids, phenolic acids, quinones, lipids, and others). A total of 151 sensory trait-correlated metabolites were obtained from the key modules via WGCNA. The majority of the 36 amino acids and derivatives, 32 others (including sugars, ketones, aldehydes, alcohols, and vitamins), 21 flavonoids, 13 phenolic acids, 13 alkaloids, 9 terpenoids, 9 nucleotides and derivatives, 8 organic acids, 4 lignans and coumarins, 3 lipids, 2 tannins, and 1 quinone were analyzed. On this basis, six significant metabolic pathways were analyzed, including nucleotide metabolism, purine metabolism, biosynthesis of alkaloids derived from histidine and purine, biosynthesis of phenylpropanoids, and anthocyanin biosynthesis. This study identifies changes in the sensory properties of candied preserved prune and their association with metabolite composition, providing a scientific basis for future efforts to improve French plums’ processing quality. An in-depth understanding of metabolic changes during processing could provide guidance for improving the production process of French plums, enhancing product quality, and developing new products with specific health benefits. These findings will not only help to enhance the market competitiveness of preserved French plums but also help to fulfill consumer demand for healthy and tasty food.

## Figures and Tables

**Table 1:** French plum sensory evaluation form.

Type	Quality	Score
Appearance	The skin is not damaged, and the appearance is full	16–20
	The epidermis is basically undamaged, and the appearance is partially full	10–15
	The skin is broken, and the appearance is shriveled	0–9
Taste	Sweet and sour (delicious); soft and hard (moderate)	16–20
	Sweet or sour; soft or hard in texture	10–15
	Too sweet or too light; hard texture (difficult to chew)	0–9
Color	The color is uniform, shiny, and crystal clear	16–20
	The color is uniform; general luster; slightly transparent	10–15
	Brown color; dull	0–9
Fragrance	It has the characteristic taste and smell of a prune	16–20
	Prune fragrance is weak	10–15
	No aroma; odor	0–9
Morphology	No return sand crystallization; no sugar flow	16–20
	There is basically no crystallization and sugar flow in sand	10–15
	Sand crystallization and sugar flow were observed	0–9

## Data Availability

The data presented in this study are available on request from the corresponding author.
